# Foxc2 regulates osteogenesis and angiogenesis of bone marrow mesenchymal stem cells

**DOI:** 10.1186/1471-2474-14-199

**Published:** 2013-07-02

**Authors:** Wulin You, Hongwei Gao, Lihong Fan, Dapeng Duan, Chunsheng Wang, Kunzheng Wang

**Affiliations:** 1 Department of Orthopedics, The Second Affilliated Hospital of Xi’an Jiaotong University, Xiwu Road, Xi’an, Shaanxi Province, 710004, China; 2 Department of Orthopedics, The Ninth Hospital of Xi’an Jiaotong University, Xi’an, Shaanxi Province, 710000, China

**Keywords:** Bone marrow mesenchymal stem cells, Foxc2, Osteogenesis, Angiogenesis, Differentiation

## Abstract

**Background:**

The Forkhead/Fox transcription factor Foxc2 is a critical regulator of osteogenesis and angiogenesis of cells. Bone marrow mesenchymal stem cells (BMSCs) have the capacity to differentiate into osteoblasts, chondrocytes, adipocytes, myocytes and fibroblasts. The present study investigates the role of Foxc2 overexpression in osteogenesis and angiogenesis of BMSCs in vitro.

**Methods:**

BMSCs were isolated from SD rat femurs and tibias, and characterized by cell surface antigen identification and osteoblasts and adipocytes differentiation. The cells were transfected with lentiviral Foxc2 (Lv-Foxc2) or green fluorescent protein (Lv-GFP). Seventy hours later, Foxc2 expression was observed using real time-PCR and Western blot. The transfected cells were stained with Alizarin red S or alkaline phosphatase (ALP) after osteogenic induction. Meanwhile, the expression levels of osteocalcin (OCN), Runt-related transcription factor 2 (Runx2), vascular endothelial growth factor (VEGF) and platelet-derived growth factor-β (PDGF-β) were measured by real time-PCR, Western blot and immunostaining.

**Results:**

Results of cell characterization showed that the cells were positive to CD44 (99.56%) and negative to CD34 (0.44%), and could differentiate into osteoblasts and adipocytes. Foxc2 overexpression not only increased the numbers of mineralized nodes and ALP activity, but also enhanced the expressions of Runx2, OCN, VEGF and PDGF-β in transfected BMSCs after osteogenic induction. The effects of Foxc2 on osteogenesis and angiogenesis were significantly different between Lv-Foxc2 transfected BMSCs and Lv-GFP transfected BMSCs (P<0.05). In addition, the MAPK-specific inhibitors, PD98059 and LY294002, blocked the Foxc2-induced regulation of BMSC differentiation.

**Conclusions:**

Foxc2 gene is successfully transfected into BMSCs with stable and high expression. The overexpression of Foxc2 acts on BMSCs to stimulate osteogenesis and angiogenesis. The effect of Foxc2 on angiogenesis of the cells is mediated via activating PI3K and ERK.

## Background

Bone formation is a temporally controlled, multistep process, and the equilibrium between bone formation by osteoblasts and bone resorption by osteoclasts is central in the maintenance of bone integrity [1]. Both osteoblasts and adipocytes share a common progenitor derived from bone marrow mesenchymal stem cells (BMSCs), and bone loss is associated with an expansion of adipose tissue in bone marrow [2]. Angiogenesis is a process involving endothelial cell proliferation and migration, and vascular tube formation; it always accompanies osteoblast differentiation and bone formation [3,4].

BMSCs have been a focus of research in stem cell-based tissue engineering during the last decade. This is due to their capacity to differentiate into osteoblasts, chondrocytes, adipocytes, myocytes and fibroblasts. Studies have shown that BMSCs have the potential to promote angiogenesis, which makes them an ideal cell type in engineering vascularized tissue [5,6]. The cells can also differentiate into osteoblasts and contribute to bone formation [7-9]. The osteoblast differentiation is mediated by various signaling molecules and transcriptional regulators, such as Wnt/beta-catenin, Notch and Hedgehog signaling pathways, and Runx2 and Osterix transcriptional factors [10,11].

Foxc2 is a member of the family of winged helix/forkhead transcription factors and is known to be expressed mainly in mesenchymal tissue [12]. Fox protein family members are important for a wide spectrum of biological processes, including metabolism, development, differentiation, proliferation, apoptosis, migration, invasion and longevity [13]. It has been reported that Foxc2-deficient mice display defective formation of the aortic arches, multiple craniofacial bones and vertebral columns, indicating an essential role of the gene in the normal development of the axial skeleton and aortic arches in mice [14,15]. Besides, Foxc2 inhibits white adipocyte differentiation by suppressing the PPARγ-induced adipogenic gene expression [16], and it regulates angiogenesis by regulating the expressions of various genes involved in the angiogenic process through activating their promoters via Fox-binding elements (FBEs) [17,18]. In addition, recent studies demonstrate that haplodeficiency of Foxc2 may result in impaired formation of tumor blood vessels as well as reduced tumor growth, and thereby provide evidence for the association of Foxc2 with the metastasis and angiogenesis of tumors [19,20]. However, it remains unclear how this transcription factor functions during osteogenesis and angiogenesis.

The present study was aimed to determine the role of Foxc2 overexpression on osteogenesis and angiogenesis of BMSCs. The molecular mechanisms of Foxc2 transcriptional regulation were also investigated.

## Methods

### Materials

Lentivirus packaging system, including plasmid pGC-FU, pHelper 1.0, pHelper 2.0 and plasmid Foxc2, was purchased from Shanghai GeneChem Co., China. Alizarin red S and Oil Red O staining kits were obtained from Winchem Industrial Co. Ltd., China. Dulbecco’s modified Eagle’s medium (DMEM) and fetal bovine serum (FBS) were purchased from GIBIC, USA. Antibodies against Foxc2, anti-CD44, anti-CD34 and β-actin were bought from Santa Cruz Biotechnology, USA. Antibodies against OCN, VEGF and PDGF-β were purchased from Abcam, USA. Antibodies against Runx2, ERK and PI3K were purchased from Novus, USA.

### Methods

#### Isolation, culture, and verification of cells

Six male SD rats, weighing 150 ± 2 g, were obtained from the Laboratory Animal Center in Medical College of Xi’an Jiaotong University. All animal protocols followed the recommendations and guidelines of the National Institutes of Health and were approved by the Animal Care and Use Committee at Xi’an Jiaotong University. BMSCs were isolated following the method described in [21]. In brief, the femurs and tibias of the rats were removed. Muscles and extraosteal tissues were trimmed. Bone marrow cells were flushed and centrifuged on a 1.073 g/mL Percoll density gradient (Pharmacia, St. Louis, USA). The cells were washed twice with PBS, seeded into 25 cm^2^ cell culture flasks, and cultivated in L-DMEM supplemented with 10% FBS and 20 mg penicillin-streptomycin/ml. Finally, the cultures were incubated in a humidified atmosphere of 95% air and 5% CO_2_ at 37°C. The medium was changed on the 4th day of culture and every 3 days after that. When the cells became subconfluent, usually after 7–10 days of culture, cells were detached using trypsin/EDTA (0.25% trypsin and 0.02% EDTA) (Sigma), concentrated by centrifugation at 1000 rpm for 5 min and then suspended in medium. An analysis of cell surface molecules, CD44 and CD34, was conducted on passage 3 cultures using flow cytometry. Osteoblasts and adipocytes differentiation was detected with Alizarin red S and Oil Red O staining kits under different induction conditions.

### Differentiation protocol and cell treatment

The transfected BMSCs were seeded in 24-well plates at a density of 1×10^6^ cells/well. Osteogenic differentiation was induced under an osteogenic condition [DMEM supplemented with 10% FBS, 0.1 μM dexamethasone (Sigma), 50 μM ascorbate acid (Sigma), and 10 mM β-glycerophosphate sodium (Sigma)] and an adipogenic condition [DMEM supplemented with 10% FBS, 200 μM indomethacin (Sigma), 1 μM dexamethasone, 0.5 mM isobutyl methylxanthine (Sigma), and 0.5 μg/mL insulin (Sigma)], respectively. For an analysis of the mechanisms involved in Foxc2-mediated regulation of differentiation, PD98059 (Sigma) at 20 μM and 50 μM, and LY294002 (Sigma) at 10 μM and 50 μM were added in the differentiation medium.

### Lentiviral production and transfection

A lentiviral vector expressing human Foxc2 and a control vector expressing GFP were purchased from Shanghai GeneChem Co., China. The lentiviruses were propagated in 293T cells and the final titer of virus was 2×10^8^ TU/ml. The multiplicity of infection (MOI) was defined as the ratio of the total number of PFU used in a particular infection to the total number of cells to be transfected.

Third-passage cells were transfected with lentivirus vectors containing either GFP or Foxc2 overnight at MOIs of 10, 50, 100 and 200 plaque-forming units (PFU)/cell. The efficiency of transfection was evaluated under a fluorescence microscope, followed by replacement of the culture medium with fresh L-DMEM supplemented with 10% FBS. Cells were used for the following experiments 72 hours after transfection.

### Cell proliferation

The transfected BMSCs were seeded in 96-well plates at a density of 5× 10^4^ cells/well and cultured for 7 days. MTT assay (Sigma) was performed following the manufacturer's instructions to detect cell proliferation.

### Western blot analysis

Cells were washed with cold PBS (pH 7.0) and lysed at 4°C in lysis buffer [50 mM Tris–HCl (pH 7.4), 150 mM NaCl, 20 mM EDTA, 1% Triton X-100, 1% sodium deoxycholate, 0.1% SDS, and protease inhibitors]. The samples were heated at 95°C for 5 min and loaded on 12% SDS-PAGE gels and transferred onto methanol-activated PVDF membranes (Millipore, Bedford, MA, USA). After blockage with 5% nonfat milk in TBST (Tris-buffered saline plus 0.1% Tween 20), the membranes were incubated with primary antibodies against Foxc2, OCN, Runx2, VEGF, PDGF-β, ERK, PI3K and β-actin, followed by incubation with the corresponding secondary antibodies. The bands were visualized by using an ECL chemiluminescence kit (Santa Cruz, CA).

### Real time-PCR analysis

Total RNA of cells was isolated using TRIZOL reagent (Invitrogen) according to the manufacturer’s instructions. After reverse transcription reaction, template DNA was used in gene-specific PCR for Foxc2, OCN, Runx2, VEGF and PDGF-β. The primer sequences used for this analysis are listed in Table 1. Glyceraldehyde 3-phosphate dehydrogenase (GAPDH) served as a housekeeping gene. The conditions of real time-PCR were as follows: 40 cycles at 94°C for 5 s and 60°C for 34 s. Dissociation stage was added to the end of amplification procedure. There was no nonspecific amplification determined by the dissolve curve.

**Table 1:** Primer oligonucleotide sequences used for real time-PCR

**Gene**	**Forward primer 5′-3′**	**Reverse primer 5′-3′**
GAPDH	CCACTTTGTGAAGCTCATTTCCT	TCGTCCTCCTCTGGTGCTCT
Foxc2	CGCCTAAGGACCTGGTGAAG	GGAAGCGGTCCATGATGA
OCN	CCACCCGGGAGCAGTGT	GAGCTGCTGTGACATCCATACTTG
Runx2	CCTTCCACTCTCAGTAAGAAGA	TAAGTAAAGGTGGCTGGATAGT
VEGF	GGCTCACTTCCAGAAACACG	GTGCTCTTGCAGAATCTAGTGG
PDGF-β	CTGCCCACAGCATGATGAGGATTGAT	GCCAGGATGGCTGAGATCACCAC
CXCR4	GGTCTGGAGACTATGACTCC	CACAGATGTACCTGTCATCC

### Immunostaining

BMSCs were fixed and treated with 50 μg/ml 4, 6-diamidino-2-phenyl-indol dihydrochlor-ide (DAPI) for nuclear staining two weeks after transfection. The cells were then stained with OCN, Runx2, VEGF and PDGF-β visualized with a TRITC-conjugated secondary antibody. The primary antibodies were diluted 1:100. Controls included staining without primary antibodies. Fluorescence images were obtained using a fluorescence microcope (fluoview 400, Olympus).

### ALP and Alizarin red S staining

After 2 weeks of transfection, ALP staining was performed using a ALP staining kit (Renbao, Shanghai, China) following the procedures provided by the manufacturer, and ALP activity was determined by the conversion of a colorless p-nitrophenyl phosphate (Pnpp, Sigma, St. Louis, USA ) to a colored p-nitrophenol [22]. For Alizarin red S staining, cells were fixed in 10% formalin and stained with 2% Alizarin red S (Ph 4.0) solution.

### Statistical analysis

Unless otherwise specified, results were presented as mean ± standard deviation (SD). Statistical analysis was performed using Student’s t-test. P<0.05 was considered statistically significant.

## Results

### Characterization of rat BMSCs

**Figure 1:**
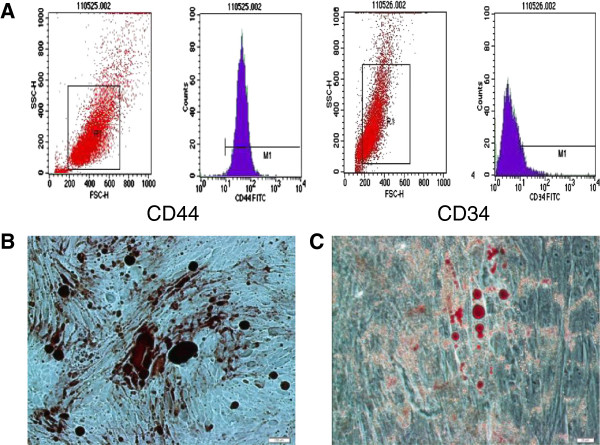
**Characterization of rat BMSCs. (A)** The results of cell surface antigen detection showed that BMSCs were positive to CD44 (99.56%) and negative to CD34 (0.44%). **(B)** The mineralization nodes were monitored by Alizarin red S staining. **(C)** Fat droplets formation was monitored by Oil Red O staining.

CD44 and CD34 were chosen as markers flow cytometry. BMSCs were successfully expanded 3–4 days after initial seeding, and rapidly expanded into colonies of confluent spindle cells at 10–14 days. The third-passage cells were incubated with antibodies of both CD44 and CD34 (Figure 1A). Results showed that the cells were positive to CD44 (99.56%) and negative to CD34 (0.44%). Matrix mineralization and fat droplet were visualized 10 days after Alizarin red S staining and Oil Red O staining (Figure 1B, C). According to the minimal criteria represented by the Mesenchymal and Tissue Stem Cell Committee of the International Society for Cellular Therapy [23], the cultured cells were effectively BMSCs.

### Foxc2 transfection

**Figure 2:**
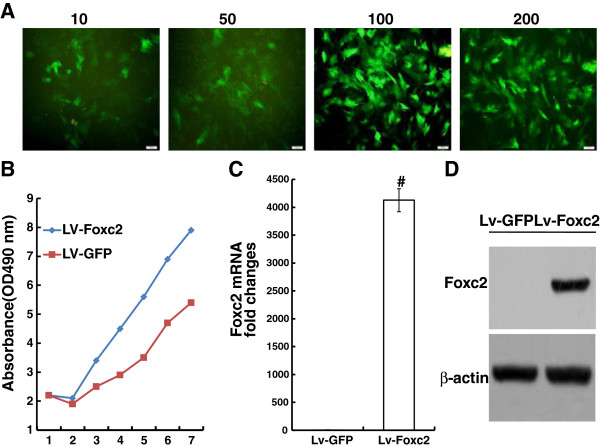
**Expression of GFP and Foxc2 genes after transfection. (A)** Transfection efficiency at various MOIs (10, 50, 100 and 200) 72 hours after transfection (×100). **(B)** Cell proliferation after transfection in the Lv-Foxc2 and Lv-GFP groups detected using MTT assay. **(C)** Foxc2 gene expression measured by real-time PCR 72 hours after transfection. GAPDH was used as a housekeeping gene. **(D)** Foxc2 protein expression evaluated by western blot 72 hours after transfection. Results were expressed as mean±SD of triplicate experiments. #p<0.01 vs. control.

When BMSCs grew to 80% confluence, the cells were transfected with Lv-GFP for 72 h at various MOIs (10, 50, 100 and 200). The efficiency of gene transfection was determined by examining GFP-positive cells under a fluorescence microscope. The percentages of GFP-positive cells at different MOIs were 27.3±1.5%, 73.4±1.6%, 91.2±1.8% and 90.8±1.5%, respectively (Figure 2A). The MTT assay data indicated that cells overexpressing Foxc2 proliferated significantly (Figure 2B).

Real time-PCR and Western blot demonstrated that Foxc2 gene was stably expressed in cells 72 h post-transfection (Figure 2C, D). Foxc2 expression was significantly higher in Lv-Foxc2 transfected cells than in Lv-GFP transfected cells (P < 0.05).

### Foxc2 enhanced osteogenesis

**Figure 3:**
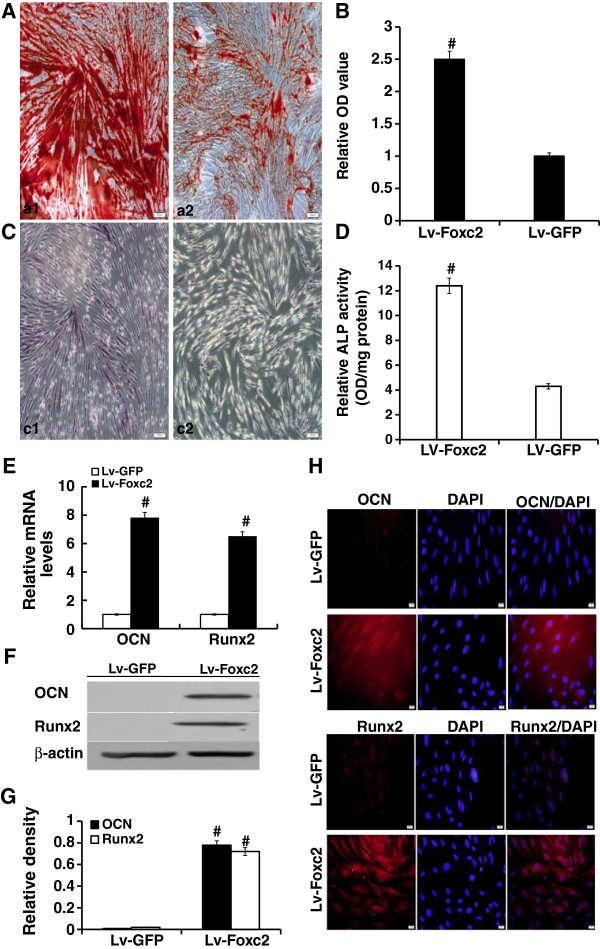
**The effect of Foxc2 on osteogenic differentiation. (A**, **B** and **C)** BMSCs were transfected with Lv-GFP or Lv-Foxc2 for 2 weeks. Cells stained with Alizarin red S (**A** and **B**, a1 presents the Lv-Foxc2 group, and a2 presents the Lv-GFP group). Cells stained with ALP (**C**, c1 presents the Lv-Foxc2 group and c2 presents the Lv-GFP group). **(D)** ALP activities in BMSCs. **(E)** Expressions of CON and Runx2 mRNA detected by real-time PCR 72 hours after transfection. The relative density of each gene was statistically analyzed. #p<0.01 vs. control. **(F** and **G)** CON and Runx2 expressions examined by Western blot 72 hours after transfection. The results were indicated by the ratio of band intensity of CON or Runx2 with β-actin. **(H)** Immunostaining for OCN and Runx2 (Texas-Red) with nuclear counterstained (DAPI-blue) 2 weeks after transfection (× 400). The results were expressed as mean±SD of triplicate experiments. #p<0.01 vs. control.

Alizarin red S staining showed that the area of positive staining of Lv-Foxc2 transfected cells was nearly 2.5 folds compared with that of the Lv-GFP transfected (Figure 3A, B). ALP expression was evidently observed in Lv-Foxc2 transfected cells after 2 weeks of induction (Figure 3C), and the ALP activity increased to nearly three folds of that in Lv-GFP transfected cells (Figure 3D). Similar expressions of osteoblast differentiation markers, OCN and Runx2, were observed in the two groups of cells (Figure 3E-H). These results demonstrated that Foxc2 overexpression enhanced osteogenesis of BMSCs.

### Foxc2 regulated angiogenesis via activating ERK and PI3K

**Figure 4:**
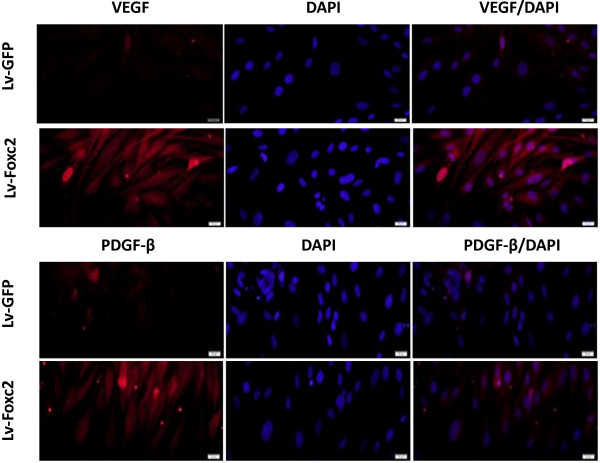
Immunostaining for VEGF and PDGF-β (Texas-Red) with nuclear counterstained (DAPI-blue) 2 weeks after transfection (× 400).

**Figure 5:**
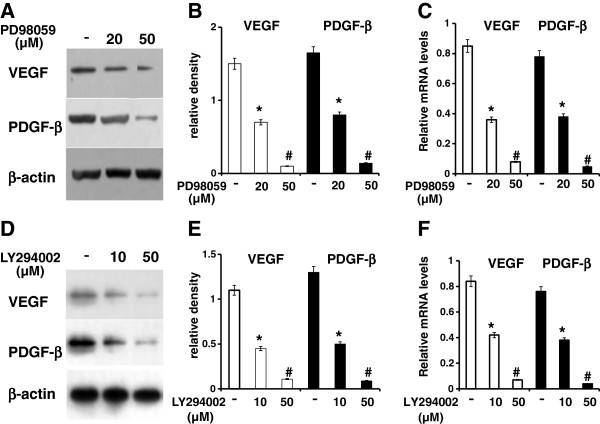
**Effect of blockade of ERK or PI3K on the expressions of angiogenic makers.** Cells overexpressing Foxc2 were cultured in osteogenic induction medium in the absence or presence of PD98059 **(A**, **B**, **C)** or LY294002 **(D**, **E**, **F)**. Western blot results of VEGF or PDGF-β expression were indicated by the ratio of band intensity of VEGF or PDGF-β with β-actin **(A**, **B**, **D**, **E)**. The results were expressed as mean±SD of triplicate experiments. Real time-PCR analysis of VEGF or PDGF-β was performed **(C**, **F)**. The relative mRNA level of each gene was quantitated and statistically analyzed. *p<0.05 vs. control; #p<0.01 vs. control.

Results of real-time PCR, Western blot and immunostainning showed that the angiogenic markers, VEGF and PDGF-β, were highly expressed in Lv-Foxc2 transfected cells while rarely expressed in Lv-GFP transfected cells (Figure 4). This suggested that Foxc2 hyper-activation might provide the transfected cells a pro-angiogenetic inclination. The VEGF-activated intracellular pathways, PI3K and ERK, can modulate the transcriptional activity of Foxc proteins in Dll4 and Hey2 induction. To investigate the role of the two signaling pathways in the regulation of angiogenesis by Foxc2, the transfected BMSCs were induced to differentiate with or without adding the inhibitors of ERK or PI3K. The results showed that the addition of the specific inhibitor of ERK or PI3K, namely, PD98059 (20 and 50 μM) or LY294002 (10 and 50 μM), led to a decrease in the gene expressions of VEGF and PDGF-β in cells overexpressing Foxc2 (Figure 5A-F). This indicated that inhibition of ERK or PI3K interfered with the regulation of Foxc2 in BMSC differentiation.

**Figure 6:**
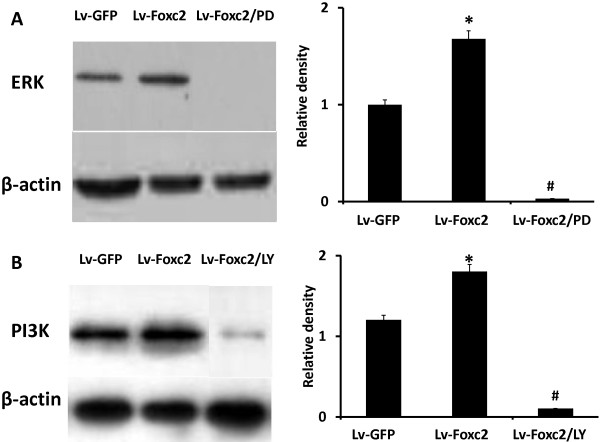
**Foxc2 overexpression induces activation of ERK and PI3K in BMSCs. (A**, **B)** BMSCs were transfected with Lv-GFP, Lv-Foxc2 or Lv-Foxc2 following pretreatment with 50 μM PD98059 or 10 μM LY294002 for 1 h. Cell lysates harvested after 2 weeks of culture were analyzed by SDS-PAGE and Western blot with anti ERK or anti-PI3K antibody. The result of ERK or PI3K expression was indicated by the ratio of band intensity of ERK or PI3K with β-actin. *p<0.05 vs. Lv-GFP group; #p<0.01 vs. group.

The levels of ERK and PI3K were higher in the cells transfected with Lv-Foxc2, indicating that Foxc2 overexpression activated the two signaling pathways in differentiation medium, but the activation was significantly abrogated by pre-treating cells with PD98059 or LY294002 (Figure 6A, B).

## Discussion

In this study, we demonstrate that Foxc2 overexpression enhances osteogenesis of BMSCs and provides the cells a major pro-angiogenetic inclination. This is consistent with the research of Arnold Caplan, et al. [24]. Our results also show that Foxc2 plays an important regulative role in angiogenesis via activating ERK or PI3K signaling pathway.

The Forkhead protein Foxc2 has emerged as an important regulator of epithelial-to-mesenchymal transitions (EMTs). In the execution of EMT, many genes involved in cell adhesion, mesenchymal differentiation, cell migration, and cell invasion are transcriptionally altered. The functional loss of E-cadherin in an epithelial cell is considered a hallmark of EMT. Many EMT-inducing transcription factors, including Snail, Slug, dEF1, ZEB2, Twist1, Foxc2 and Goosecoid, can repress E-cadherin directly or indirectly when overexpressed [25]. EMT-derived cells share many properties with mesenchymal stem cells (MSCs), while conversely, MSCs express some EMT-associated genes, such as Snail and Foxc2. Similar to MSCs, EMT-derived cells can also differentiate into mature osteoblasts, adipocytes and chondrocytes [26]. Moreover, EMT is an important source for the accumulation of carcinoma associated fibroblasts [27]. It has been proved that the differentiation of epithelial cells is correlated with increased levels of cytoplasmic Foxc2, whereas the dedifferentiation is associated with decreased Foxc2 levels [28]. However, the function of Foxc2 in osteoblast differentiation and angiogenesis has been rarely studied.

We have found in our study that Foxc2 is highly expressed in BMSCs after transfection, and the Foxc2 hyperexpression promotes cell viability (Figure 2). We also reconfirm that Foxc2 enhances the differentiation of BMSCs into osteoblasts (Figure 3). The mechanisms of Foxc2-regulated osteogenesis are still not completely understood. It was reported that the activation of canonical Wnt-β-catenin signals might be involved in the Foxc2-mediated osteoblast differentiation [29]. In addition, Su Jin Park et al. found that Foxc2 was a downstream target of well known anabolic systemic hormones such as BMP2 and PTH, and that Foxc2 promoted osteoblastogenesis by regulating the survival, proliferation and differentiation of osteoblasts through the up-regulation of integrin β1 [30].

Foxc2 also plays an important role in vascular endothelial development. It was reported that Foxc2 acted upstream of Notch signaling in arterial gene expression by directly regulating the Dll4 promoter via a Fox-binding element [31,32]. Bone marrow-derived cells transfected with Foxc2 caused an increased cellular mobility through the up-regulation of CXCR4 [33]. This increase in cellular mobility due to Foxc2 overexpression has been experienced in other two separate studies, in which the action of Foxc2 was linked to the regulation of two different proteins, p120-catenin and β3 integrin [34,35]. p120-catenin is a regulator of E-cadherin, which promotes cell-cell adhesion at adherens junctions. Loss of p120-catenin expression results in the destabilization of the E-cadherin complex, which is a critical step in invasion and metastasis [34]. Physiologically, Foxc2 is activated by hypoxia and VEGF. It acts on specific ligand proteins, including p120-catenin and β3 integrin, to destabilize them and promote cellular releasing from their environment. It induces CXCR4 transcription, allowing cells to migrate to the site of injury or hypoxia following a chemokine (CXCL12) gradient. High CXCR4 levels in tumors are linked to poor survival [35].

We have found that Foxc2 overexpression enhances the expressions of angiogenic factors such as VEGF and PDGF-β (Figures 4, 5), and increases the protein levels of ERK and PI3K. The ERK or PI3K inhibitor, PD98059 or LY294002, attenuates the Foxc2-mediated enhancement of angiogenic factors (Figures 5, 6). It has been found that VEGF-activated PI3K and ERK pathways modulate the transcriptional activation of Dll4 and Hey2 genes by Foxc proteins [36]. A recent research shows that VEGF-stimulated PI3K and ERK pathways modulate the transcriptional activity of Foxc2 for arterial gene expression in endothelial cells [37]. Consequently, functional interaction between VEGF signaling and Foxc2 may take place in some aspects of blood vessel formation.

In most in vitro contexts, the PI3K and ERK pathways are stimulated by VEGF together and often act in a synergistic manner. PI3K activation leads to AKT activation, which promotes the migration and survival of endothelial cells and nitric oxide production. ERK/MAPK activation promotes endothelial cell proliferation. Nonetheless, in certain endothelial culture systems, the PI3K branch antagonizes the ERK/MAPK branch [38]. The reasons for the discrepancy of the functional effects of ERK and PI3K are unclear.

MSCs, when are on a stiff substrate and in large numbers, tend to spontaneously differentiate over time into osteoblasts, and this process may be speeded by the overexpression of an important transcriptional factor, Foxc2 [39]. However, many data suggest that the up-regulation of Foxc2, or its transfection, leads to an increase of cellular mobility often linked with progression, invasion and angiogensis of tumor [19,20,33]. Thus, the clinical safety of Foxc2-based therapy should still be verified.

## Conclusions

Taken together, this work examines the effects of Foxc2 on the commitment of SD rat BMSCs into the osteogenic and angiogenic lineages in vitro. Our results demonstrate that Foxc2 overexpression acts on the transfected BMSCs to enhance the expressions of osteogenic makers and provide the cells a pro-angiogenetic inclination. Furthermore, it is likely that ERK and PI3K signaling pathways are involved in the Foxc2-mediated regulation of angiogenetic inclination.

Future research on the function of Foxc2 in osteogenesis and angiogenesis can be conducted on some novel cell models to study its effects on different mesenchymal-related differentiation processes.

## Abbreviations

BMSCs: Bone marrow mesenchymal stem cells; Foxc2: Forkhead box C2; GFP: Green fluorescent protein; ALP: Alkaline phosphatase; OCN: Osteocalcin; Runx2: Runt-related transcription factor 2; VEGF: Vascular endothelial growth factor; PDGF-β: Platelet-derived growth factor-β; PI3K: Phosphoinositide-3-kinase; ERK: extracellular signal regulated/mitogen activated protein kinase; PPARγ: Peroxisome proliferator activated receptor gamma 2; CXCR4: C-X-C chemokine receptor type 4; Dll4: Delta-like 4; FBEs: Fox-binding elements; DMEM: Dulbecco’s modified Eagle’s medium; FBS: Fetal bovine serum; MOI: Multiplicity of infection; GAPDH: Glyceraldehyde 3-phosphate dehydrogenase; DAPI: 4, 6-diamidino-2-phenyl-indol dihydrochlor-ide; EMTs: Epithelial-to-mesenchymal transitions.

## Competing interests

The authors declare that they have no competing interests.

## Authors’ contributions

WY and KW are the principal investigators. WY and KW contributed to conception and design, acquisition, analysis and interpretation of data and were involved in drafting the manuscript. WY, HG and DD carried out the experiments. LF and CW were responsible for the statistical analysis and evaluation of the data. All authors read and approved the final manuscript.

## Pre-publication history

The pre-publication history for this paper can be accessed here:

http://www.biomedcentral.com/1471-2474/14/199/prepub
